# Patients and informal caregivers' experience of surgical and transcatheter aortic valve replacement: Real‐world data contributing to establish value‐based medicine in Denmark

**DOI:** 10.1002/clc.23166

**Published:** 2019-03-14

**Authors:** Liesbeth Rosseel, Gintautas Bieliauskas, Bente B. Brodersen, Peter S. Olsen, Lars Søndergaard, Ole De Backer

**Affiliations:** ^1^ The Heart Center, Rigshospitalet Copenhagen Denmark

**Keywords:** aortic valve stenosis, heart valve prosthesis implantation, quality of life, transcatheter aortic valve replacement

## Abstract

**Background:**

The concept of value‐based medicine (VBM) is increasingly implemented in therapeutic decision‐making processes, but only few data on patient‐perceived values are available in the field of aortic stenosis treatment.

**Hypothesis:**

This study aimed to deliver data on patient‐perceived values and health‐related quality of life (HR‐QoL) following surgical aortic valve replacement (SAVR) and transcatheter aortic valve replacement (TAVR) in a real‐world, all‐comers patient population.

**Methods:**

Questionnaires were sent to 637 patients who had undergone elective AVR 12 to 24 months earlier in the period September 2015 to August 2016. The questionnaires were specifically designed to assess physical and mental impact of the entire AVR process on patients and their nearest relative and to capture HR‐QoL.

**Results:**

Questionnaires were completed by 429 patients (SAVR: N = 265; TAVR: N = 164). Both physical and mental impact of the intervention and its recovery period were experienced more stressful by SAVR as compared with TAVR patients. Also, nearest relatives of SAVR patients experienced the entire process mentally more stressful and enduring than relatives of TAVR patients. In both groups, 10% of patients reported no change in HR‐QoL, whereas HR‐QoL improved in 76% vs 83% (*P* = 0.092) and worsened in 14% vs 7% (*P* = 0.040) of the SAVR and TAVR populations, respectively.

**Conclusion:**

The perioperative experience tends to be more stressful for SAVR as compared with TAVR patients; however, HR‐QoL finally improves to a similar degree in both groups. Given the increasing importance of VBM, patient‐perceived values will have to be considered in future decision‐making processes, both at individual and public policy‐making level.

## 1 INTRODUCTION

The standard of care for patients with severe, symptomatic aortic valve stenosis (AS) has radically changed over the past decade. Aortic valve replacement (AVR) is no longer an operation that is approached solely through a median sternotomy. Based on data from large randomized trials, transcatheter aortic valve replacement (TAVR) has become an established therapeutic option for patients with severe AS who are at increased risk for conventional surgical AVR (SAVR).^1^, ^2^, ^3^, ^4^ In recent years, the TAVR technology is also increasingly used to treat patients with a lower risk profile. In the PARTNER‐II, SURTAVI, and NOTION trials, robust randomized data demonstrated the equivalence—and even net superiority when performed via transfemoral approach—of TAVR in lower risk patients.^2^, ^3^, ^4^

Evidence‐based data and medicine (EBM) are the cornerstone of global healthcare in the 21st century. Currently, the health system in Denmark is moving toward value‐based medicine (VBM), in which patient‐perceived value‐based data are integrated into the best available evidence‐based data, so that it allows clinicians to deliver higher quality patient care than EBM alone. The ultimate goal of VBM is to add quality to years based on the best possible resource utilization.^5^ Only limited data on health‐related quality of life (HR‐QoL) in SAVR and TAVR patients are available in literature—and most often, these data are based on results from the Kansas City Cardiomyopathy Questionnaire (KCCQ), an instrument used to evaluate the health status of heart failure patients. For this study, a dedicated questionnaire was composed to assess the perioperative physical and mental stress experienced by the patient and his/her nearest relative as well as the overall HR‐QoL—and this in a real‐world, all‐comers patient population undergoing elective AVR.

## 2 METHODS

### 2.1 Study population

All patients who underwent elective SAVR or TAVR at Rigshospitalet, Copenhagen (Denmark) between September 2015 and August 2016 were considered for this study. A questionnaire directed to the patient and his/her nearest relative was sent to all patients, excluding those who had expired at the time of the questionnaire mailing (September 2017). The final study population consisted of all patients responding to the questionnaire. In accordance with the institutions' policy, all patients gave written informed consent for the procedure and the use of anonymous data for research. The study was conducted in accordance with the declaration of Helsinki.

### 2.2 Procedures

All patients were discussed by a multidisciplinary Heart Team and found eligible for SAVR or TAVR; also, the indication for a concomitant procedure (coronary revascularization, aortic root replacement, and/or other valve surgery) was discussed at this meeting. Procedures were performed in accordance with the local routine practice and the best clinical standards. Most TAVR procedures were performed by transfemoral approach (95%) under local anesthesia; other accesses included a transsubclavian and transapical approach. Prospective data collection involved demographic and procedural data.

### 2.3 Questionnaires

The questionnaires in this study were specifically designed to capture patients and informal caregivers' perioperative experience as well as the patients' HR‐QoL before and after AVR (Supplementary Material). The questionnaires were designed by an experienced team of transcatheter valve therapies nurses/physicians and clinical research nurses and were based on the three components of “health” as defined by the World Health Organization (WHO); that is, “a state of complete physical, mental, and social well‐being and not merely the absence of disease”. HR‐QoL is defined as “an individual's perception of the influence of an illness and its treatment on the quality of life and the functioning of an individual”.^6^, ^7^ The validity of all questions was checked by a pilot study (n = 5) to check whether all subjects interpreted the questions correctly and in the same way. Also, the social status of the patient was documented. Given the high age of the study population and the impact of the treatment, a dedicated questionnaire was also directed toward the patient's nearest relative (Supplementary Material).

### 2.4 Statistical analysis

Descriptive statistics were expressed as mean ± SD for continuous variables and as frequency and percentages (%) for discrete variables. The differences in means between groups were determined using Student's *t* test or Wilcoxon rank‐sum test, whereas *χ* ^2^ test was used to test for associations between discrete variables. A two tailed *P*‐value <0.05 was considered to indicate statistical significance. Statistical analyses were performed using commercially available software (SPSS version 24.0, IBM, Armonk, New York).

## 3 RESULTS

### 3.1 Study population

All patients undergoing elective AVR and alive in September 2017 (n = 637) were considered for this study; only non‐elective cases in the context of endocarditis or acute aortic dissection were excluded from this study. Questionnaires were completed by 429 patients (SAVR: n = 265; TAVR: n = 164), representing 63% of the initial 686 patients that had undergone elective AVR. Mortality (7%‐8%) and questionnaire response rates (67%‐68%) were similar in both populations (NS; Supporting Information Figure S1). Of all 429 returned questionnaires, 384 (90%) were also containing responses from the nearest relative.

All baseline characteristics of the study population, dichotomized by SAVR vs TAVR, are shown in Table S1. The SAVR population was significantly younger than the TAVR population and comprised a higher number of male and lower surgical risk patients. In accordance with the older age of the TAVR population, these patients were also more often living alone without partner. Hospitalization at intensive care unit was virtually non‐existing for the TAVR group and total hospitalization length was approximately 3× longer for the SAVR group as compared with the TAVR group (11.2 ± 9.2 vs 3.6 ± 2.4 days, respectively; *P* < 0.001; Table S1).

### 3.2 Physical and mental burden

Knowing the impact of AVR on patients in daily clinical practice, this AVR‐dedicated questionnaire specifically assessed how physically and mentally stressful the procedure and recovery period was for the patient.

**Figure 1:**
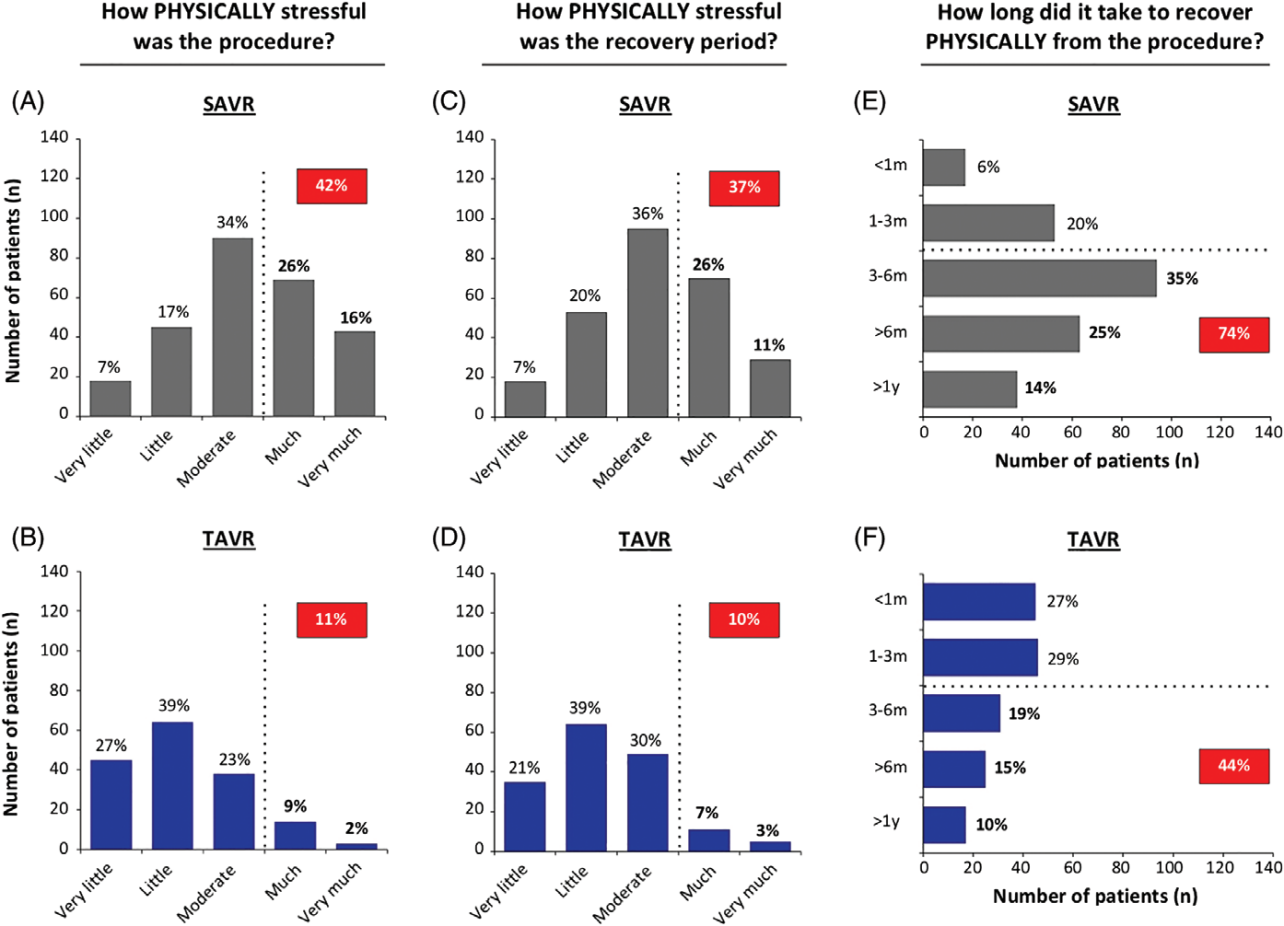
Physical stress. Bar charts showing the number of patients that evaluated the procedure (A and B) and the recovery period (C and D) as physically stressful to a certain degree (very little, little, moderate, much, very much). In addition, patients indicated how long time it took to recover physically from the procedure (E and F). SAVR, surgical aortic valve replacement; TAVR, transcatheter aortic valve replacement

**Figure 2:**
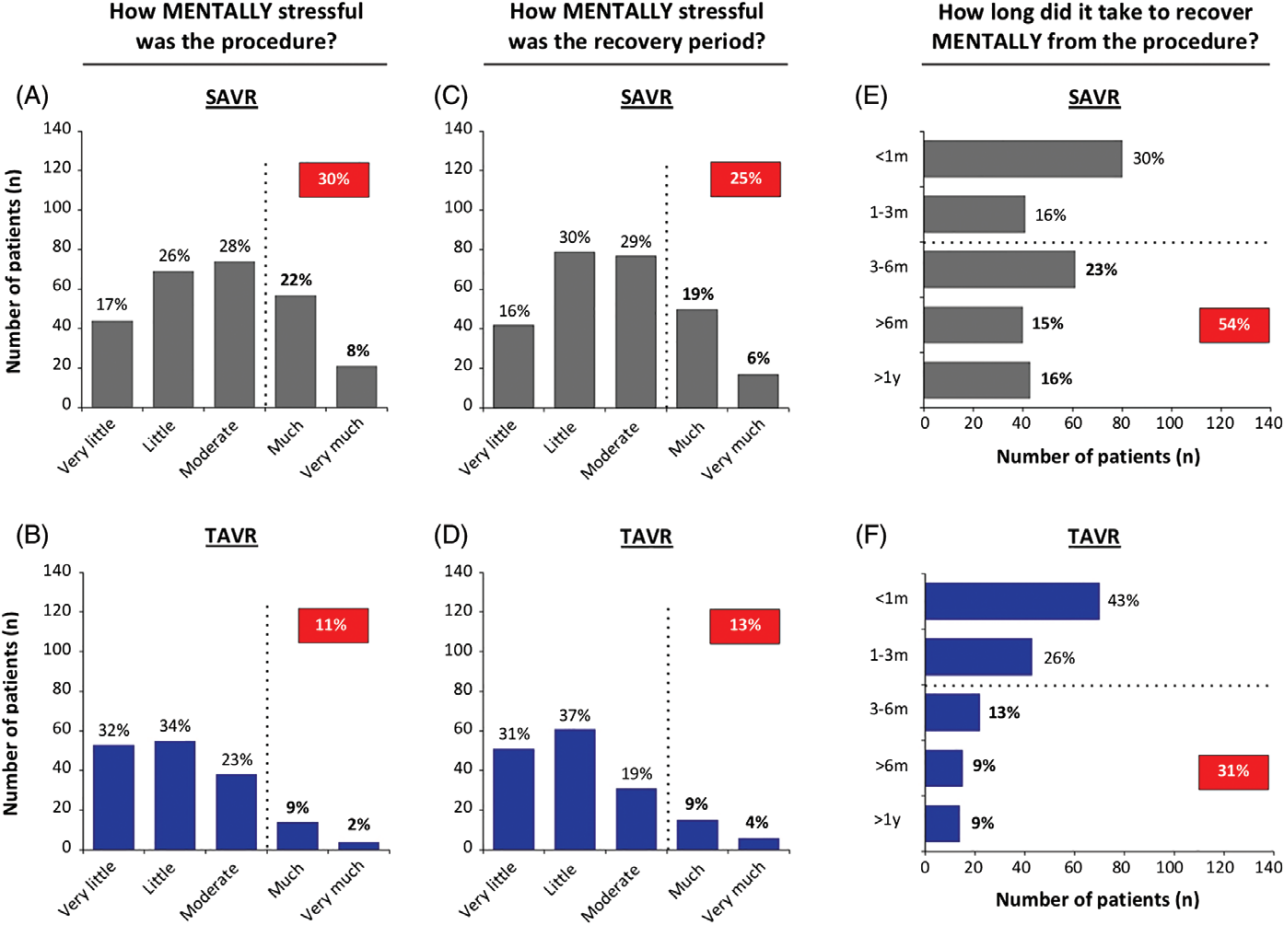
Mental stress. Bar charts showing the number of patients that evaluated the procedure (A and B) and the recovery period (C and D) as mentally stressful to a certain degree (very little, little, moderate, much, very much). In addition, patients indicated how long time it took to recover mentally from the procedure (E and F). SAVR, surgical aortic valve replacement; TAVR, transcatheter aortic valve replacement

In the SAVR group, 42% of patients reported to experience the surgical procedure as physical stressful (level ≥ much), whereas only 11% of TAVR patients experienced the procedure as physically stressful (level ≥ much; *P* < 0.001; Figure 1A,B). In accordance, 30% of SAVR patients vs 11% of TAVR patients experienced the procedure as mentally stressful (level ≥ much; *P* < 0.001; Figure 2A,B).

Also the recovery period was physically and mentally more stressful (level ≥ much) for the SAVR group as compared to the TAVR group (physically: 37% vs 10% and mentally: 25% vs 13%, respectively; *P* < 0.01; Figure 1C,D**;** Figure 2C,D). The physical recovery period was longer than 3 months for 74% of SAVR vs 44% of TAVR patients (*P* < 0.001; Figure 1E,F). The mental recovery period took longer than 3 months for 54% of patients in the SAVR group vs 31% in the TAVR group (*P* < 0.001; Figure 2E,F).

### 3.3 Overall HR‐QoL

**Figure 3:**
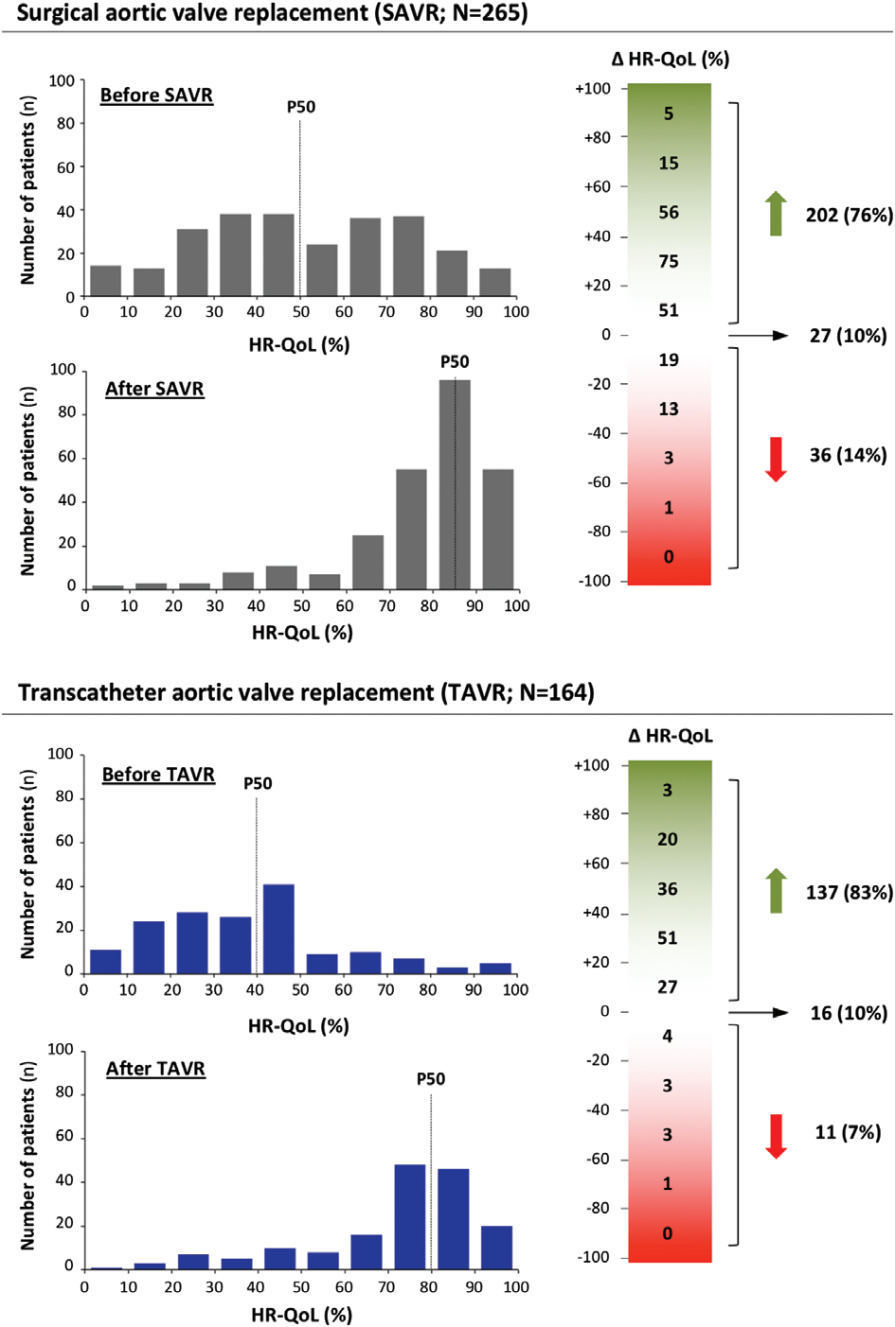
Health‐related quality of life (HR‐QoL). Schematic figure showing the reported HR‐QoL by all SAVR and TAVR patients before and after intervention; the dotted line shows the median (P50). The green‐red bar on the right indicates the number of patients that reported an improved, unchanged or worse HR‐QoL after intervention. SAVR, surgical aortic valve replacement; TAVR, transcatheter aortic valve replacement

**Figure 4:**
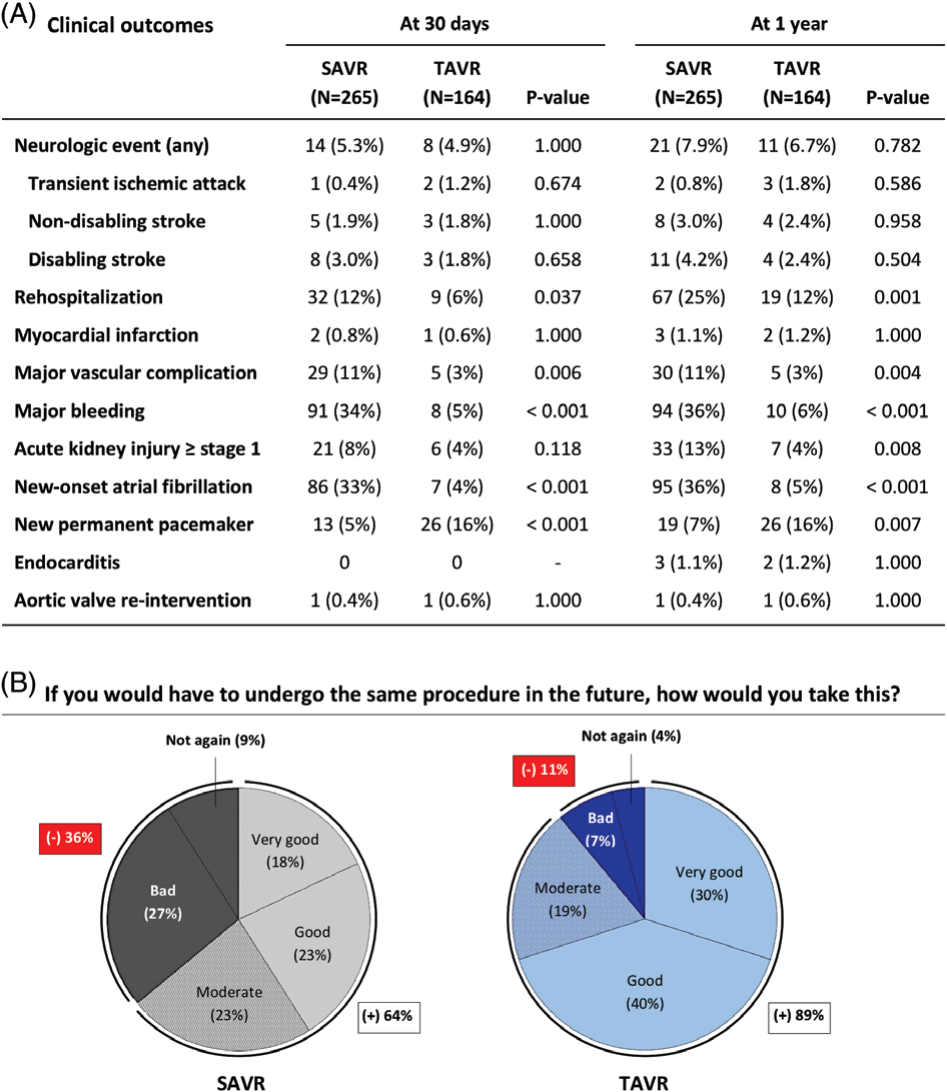
A, Clinical outcomes. Clinical outcomes at 30‐day and 1‐year as defined by the Valve Academic Research Consortium (VARC)‐2 criteria. B, Redo‐intervention. Cake diagrams indicating the percentage of patients that were standing positive (+) or negative (−) toward a possible similar intervention in the future. SAVR, surgical aortic valve replacement; TAVR, transcatheter aortic valve replacement

All patients assessed their HR‐QoL on a metric scale ranging from 0 (worst) to 100 (best), as rated at baseline before intervention and at 12 to 24 months after intervention (mean of 18 ± 4 months after AVR). The median baseline HR‐QoL reported in the SAVR group was 50% and increased to 85% after surgical intervention, whereas the median baseline HR‐QoL in the TAVR group was reported to be 40% with an increase after intervention to 80%. In both groups, 10% of patients reported no change in HR‐QoL, whereas the HR‐QoL improved in 76% vs 83% (*P* = 0.092) and became worse in 14% vs 7% (*P* = 0.040) of the SAVR and TAVR populations, respectively (Figure 3). The major reasons for reporting no improvement in HR‐QoL were increased dyspnea, fatigue or dizziness as compared with the pre‐procedural status, unhappiness about the intervention or logistics in general, stroke with neurological impairment and recurrent hospitalizations following SAVR and TAVR as well as invalidating episodes of atrial fibrillation and mental changes after cardiac surgery. A list of all collected reasons can be found in Table S2 **.** Clinical outcomes at 30 days and 1 year, as assessed according to the Valve Academic Research Consortium (VARC)‐2 criteria, are reported in Figure 4A**.** 8

Similar results were obtained when the above‐mentioned analyses were repeated for all SAVR patients excluding those that had undergone concomitant cardiac surgery (n = 195; Figure S2 A‐C) as well as for those SAVR and TAVR patients with an intermediate surgical risk (STS surgical risk score 3%‐8%; Figure S2 D‐E).

With regards to NYHA functional classification, similar tendencies as reported for HR‐QoL were observed. More patients reported NYHA class III‐IV at baseline in the TAVR group as compared to the SAVR group (61% vs 34%; *P* < 0.001) but with similar outcomes concerning functional improvement (Figure S3).

Finally, patients were asked how they would react if they had to undergo the same procedure again. In the SAVR group, 36% of patients were negative about a possible redo‐SAVR, indicated by (−) in Figure 4B, whereas only 11% of TAVR patients were negative about a possible redo‐TAVR procedure in the future.

### 3.4 Informal caregivers

**Figure 5:**
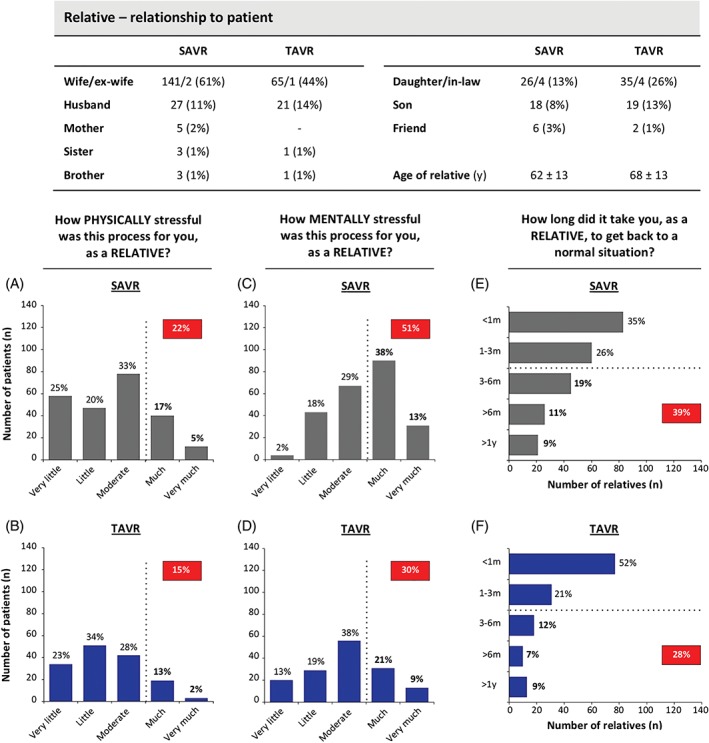
Informal caregivers. Different relationships of the informal caregivers to the patients in both groups. The vertical bar charts show the number of informal caregivers that evaluated the entire process physically (A and B) and mentally (C and D) stressful to a certain degree (very little, little, moderate, much, very much). In addition, informal caregivers indicated how long time it took to recover from the entire process (E and F). SAVR, surgical aortic valve replacement; TAVR, transcatheter aortic valve replacement

As indicated in Figure 5, the nearest relative—representing the patients' informal caregiver—was in 98% of cases a family member; most often the wife but also frequently the daughter(−in‐law) of TAVR patients. Perioperative physical stress was not significantly different for informal caregivers of SAVR and TAVR patients (level ≥ much: 22% vs 15%; *P* = 0.098). However, nearest relatives of SAVR patients experienced the process mentally more stressful as compared to relatives of TAVR patients (level ≥ much: 51% vs 30%, *P* < 0.001).

The indicated time‐to‐recover from this entire process was longer for informal caregivers of SAVR as compared with TAVR patients (>3 months: 39% vs 28% for SAVR and TAVR relatives, respectively; *P* = 0.026; Figure 5).

## 4 DISCUSSION

In light of the emerging implementation of VBM in East Denmark, we assessed—in this prospective, single‐center study—patient‐perceived perioperative values in a real‐world, all‐comers North‐European population undergoing SAVR and TAVR. In addition, we examined the physical and mental impact of the entire process on the patients' nearest relative, an often neglected but increasingly recognized member of caregiving.

### 4.1 Patient‐perceived values and HR‐QoL

As data on patient‐perceived values in the field of AVR are scarce, a dedicated questionnaire was composed to assess the perioperative physical and mental stress experienced by the patient as well as the overall change in HR‐QoL—and this in an all‐comers population undergoing elective AVR.

First, the data in this study could confirm previous reports indicating that both SAVR and TAVR lead to significant improvements in HR‐QoL. Subanalyses of the PARTNER 2 and SURTAVI trials in intermediate‐risk patients—with similar clinical outcomes as to our population—also reported substantial HR‐QoL improvement based on KCCQs with temporal trends showing a greater early benefit with TAVR as compared to SAVR.^4^, ^9^, ^10^ In a study analyzing HR‐QoL outcomes of TAVR patients based on data from the GARY (German Aortic Valve Registry), TAVR treatment was shown to improve HR‐QoL, especially in terms of mobility and usual activities (EuroQoL 5D questionnaire). The magnitude of improvement was shown to be higher in the “transvascular” as compared with the “transapical” group; thereby illustrating the importance of the degree of invasiveness of the procedure.^9^, ^11^, ^12^

In this study, unique data on patient‐perceived values were acquired by questioning patients their physical and mental experience of the entire AVR process. SAVR patients experienced the procedure and recovery period both physically and mentally more stressful than TAVR patients, needing substantially longer time to recover from the intervention.

It is important to realize that there are some key differences between this study and prior studies. First, this study introduced a new questionnaire specifically designed to assess patient‐perceived values in the context of AVR. The randomized controlled trial (RCT) substudies mentioned above typically used KCCQs, an instrument used to evaluate the health status of heart failure patients.^13^ Second, this study mainly comprised low‐to‐intermediate risk AS patients which were in 95% of TAVR cases treated in local anesthesia using a minimalistic approach—this is very different from the large RCTs comprising higher risk patients and a larger difference in TAVR approaches. Another point of discussion concerning the large RCTs has been that a significant number of patients randomized to SAVR, up to one quarter, also underwent some other form of cardiac surgery (coronary bypass, aortic root, other heart valve). Consequently, this study also comprises a sub‐analysis excluding those SAVR patients undergoing concomitant cardiac surgery; however, this did not impact the findings in this study.

Finally, as redo‐interventions are not uncommon, our questionnaire also asked the patients' position towards a possible redo‐SAVR or redo‐TAVR in the future. A significantly larger proportion of patients was negative about a possible redo‐intervention in the SAVR group as compared to the TAVR group (36% vs 11%; *P* < 0.001). Hence, we believe the same question should be asked in daily clinical practice to every single patient that needs a redo‐cardiac intervention and who has options for transcatheter treatment.

### 4.2 Heart Team meeting in the era of VBM

In the era of VBM, the Heart Team meeting as we know today will also need an update. Besides EBM, surgical risk scores and anatomical considerations, other factors such as expected resource utilization, patient experience and preference (“shared decision‐making”), social status, anticipated hospitalization length, and need for informal caregiving will have to be considered. For example, for an 80‐year‐old patient with an STS surgical risk score < 3% but living alone, TAVR would probably be the more “value‐based” therapeutic choice. Especially when choosing between equally efficient therapies such as SAVR and TAVR, additional patient‐perceived value data will increasingly influence the therapeutic decision‐making process in the future.

### 4.3 Informal caregivers

As patients who have to undergo AVR are typically elderly and the intervention is not exactly “minor”, the role of informal caregivers in the entire process should not be underestimated. Data on the experience of patients' relatives were collected in this study, showing that relatives of SAVR patients experienced the entire process mentally—but not physically—more stressful as compared to the relatives of TAVR patients.

Interestingly, after completion of this study, a policy statement from the American Heart Association was published in *Circulation* entitled “Projected costs of informal caregiving for cardiovascular disease: 2015 to 2035”.^14^ Based on these calculations, estimated informal caregiving costs for cardiovascular disease in the US reached $61 billion in 2015. By 2035, these informal costs will more than double, reaching $126 billion. The authors conclude that informal caregivers have become a “critical issue” in public policy with growing economical and organizational importance in public health.

*Study limitations.* This study has clear limitations inherent to its non‐randomized, single‐center, observational design. In addition, comparisons between both groups need to be interpreted cautiously since baseline characteristics and health status were not completely equal between both groups and sample sizes were relatively small. This study comprised a real‐world all‐comers population of patients undergoing elective AVR during 1 year in a North European region with a population of 2.8 million—this is a strength of this study; however, this may also implicate that these results may not be automatically valid globally because of cultural differences. The response rate to the survey was high and equal in both treatment groups; however, patient selection bias cannot be excluded as participation to this questionnaire study was only on a voluntary basis. Moreover, patients that had died by the time the survey was conducted are not represented in this study. Finally, this study does not allow describing temporal trends as the questionnaire was only completed at one given time after intervention.

## 5 CONCLUSION

SAVR patients experience their procedure and recovery period both physically and mentally more stressful than TAVR patients, needing substantially longer time to recover from the intervention. Also, informal caregivers of SAVR patients experience the entire process mentally more stressful than relatives of TAVR patients. Although the non‐randomized, observational design of this study calls for caution in the interpretation of the results, it is a certainty that patient‐perceived values will have to be considered in future therapeutic decision‐making processes, both at an individual and public policy‐making level.

## CONFLICT OF INTEREST

The authors have no conflict of interest to report.

## Supporting information

Appendix S1. Supplementary material

Figure S1. Study design.

Figure S2 A and B, Physical and mental stress reported by SAVR patients without concomitant surgical intervention (N = 195). Bar charts indicating the number of patients that felt the procedure and the recovery period physically and mentally stressful to a certain degree (very little, little, moderate, much, very much). In addition, patients indicated how long time it took to recover physically and mentally from the procedure. SAVR, surgical aortic valve replacement. C, Health‐related quality of life (HR‐QoL) in SAVR patients without concomitant cardiac surgery (N = 195). Schematic figure showing the reported HR‐QoL by SAVR patients without concomitant cardiac surgery, before and after intervention. The green‐red bar on the right indicates the number of patients that reported an improved, unchanged or worse HR‐QoL after intervention. SAVR, surgical aortic valve replacement. D and E, Health‐related quality of life (HR‐QoL) in intermediate risk SAVR and TAVR patients (N = 208). Schematic figures showing the reported HR‐QoL by intermediate risk (STS surgical risk score 3‐8%) SAVR and TAVR patients, before and after intervention. The green‐red bar on the right indicates the number of patients that reported an improved, unchanged or worse HR‐QoL after intervention. SAVR, surgical aortic valve replacement; TAVR, transcatheter aortic valve replacement.

Figure S3. NYHA functional classification. Schematic figure showing the reported NYHA functional classification by all SAVR and TAVR patients before and after intervention. The green‐red bar on the right indicates the number of patients that reported an improved, unchanged or worse NYHA classification after intervention, respectively. NYHA, New York Heart Association; SAVR, surgical aortic valve replacement; TAVR, transcatheter aortic valve replacement.

Table S1. Baseline characteristics

Table S2. Patients that did not report an improvement in HR‐QoL following intervention
