# Supplementary material for: Patients and informal caregivers' experience of surgical and transcatheter aortic valve replacement: Real‐world data contributing to establish value‐based medicine in Denmark
Source: Clin Cardiol. 2019 Mar 14;42(4):444–51. doi: 10.1002/clc.23166 (PMC6712343; doi:10.1002/clc.23166)
Supplement: Supplementary file 1 — Appendix S1. Supplementary material [file CLC-42-444-s001.pdf]

<u>Spørgeskema for patienten</u>

Navn: ____ **Alder:** ________________

Køn: ☐ Mand

☐ Kvinde

Social status: ☐ Bor alene

☐ Samboende/gift

☐ Andet: ____________________________

Almen helbredstilstand

1. På denne skala bedes du angive hvor god eller dårlig du vurderer din egen helbredstilstand **<u>før du fik din nye hjerteklap</u>**. Den bedste helbredstilstand du kan forestille dig er markeret med 100, og den værste helbredstilstand du kan forestille dig er markeret med 0. Angiv dette ved at <u>sætte et kryds</u> på skalaen og skrive dette tal ind i boksen.

0 10 20 30 40 50 60 70 80 90 100

Værste helbredstilstand — Bedste helbredstilstand

2. På denne skala bedes du angive hvor god eller dårlig du vurderer din egen helbredstilstand **<u>i dag – efter du fik din nye hjerteklap</u>**. Den bedste helbredstilstand du kan forestille dig er markeret med 100, og den værste helbredstilstand du kan forestille dig er markeret med 0. Angiv dette ved at <u>sætte et kryds</u> på skalaen og skrive dette tal ind i boksen.

0 10 20 30 40 50 60 70 80 90 100

Værste helbredstilstand — Bedste helbredstilstand

Symptom vurdering

3. Hvordan vil du vurdere dine symptomer ved anstrengelse **før du fik din nye hjerteklap**?

- ☐ Jeg har ingen fysisk begrænsning ved almindelig aktivitet.
- ☐ Jeg får åndenød og/eller oplever træthed ved moderat til hårdere fysisk anstrengelse, det kan fx være ved gang på trapper til mere end anden sal.
- ☐ Jeg får åndenød og/eller oplever træthed ved lettere fysisk anstrengelse, det kan fx være ved gang på trapper til mindre end anden sal, eller ved meget beskeden aktivitet, som fx at tage tøj af og på.
- ☐ Jeg får åndenød og/eller oplever træthed i hvile.

4. Hvordan vil du vurdere dine symptomer ved anstrengelse **i dag – efter du fik din nye hjerteklap**?

- ☐ Jeg har ingen fysisk begrænsning ved almindelig aktivitet.
- ☐ Jeg får åndenød og/eller oplever træthed ved moderat til hårdere fysisk anstrengelse, det kan fx være ved gang på trapper til mere end anden sal.
- ☐ Jeg får åndenød og/eller oplever træthed ved lettere fysisk anstrengelse, det kan fx være ved gang på trapper til mindre end anden sal, eller ved meget beskeden aktivitet, som fx at tage tøj af og på.
- ☐ Jeg får åndenød og/eller oplever træthed i hvile.

Fysisk helbredstilstand

5. Hvor fysisk anstrengende vurderer du at **indgrebet** var for dig?

☐ Virkelig meget ☐ Meget ☐ Moderat ☐ Lidt ☐ Slet ikke

6. Hvor fysisk anstrengende vurderer du at **efterforløbet** var for dig?

☐ Virkelig meget ☐ Meget ☐ Moderat ☐ Lidt ☐ Slet ikke

7. **Hvor lang tid** vurderer du det tog at komme dig fysisk over hele forløbet?

☐ < 1 måned ☐ 1-3 måneder ☐ 3-6 måneder ☐ > 6 måneder ☐ Jeg er ikke kommet mig fysisk endnu

Psykisk helbredstilstand

8. Hvor psykisk anstrengende vurderer du at **indgrebet** var for dig?

☐ Virkelig meget ☐ Meget ☐ Moderat ☐ Lidt ☐ Slet ikke

9. Hvor psykisk anstrengende vurderer du at **efterforløbet** var for dig?

☐ Virkelig meget ☐ Meget ☐ Moderat ☐ Lidt ☐ Slet ikke

10. Hvor **lang tid** vurderer du det tog at komme dig psykisk over hele forløbet?

☐ < 1 måned ☐ 1-3 måneder ☐ 3-6 måneder ☐ > 6 måneder ☐ Jeg er ikke kommet mig psykisk endnu

11. Hvis du skulle gennemgå samme indgreb **igen**, hvordan ville du have det med det?

☐ Meget godt ☐ Godt ☐ Neutral ☐ Dårligt ☐ Jeg vil ikke gøre det igen

12. Andre kommentarer:

____

____

____

____

____

____

____

____

____

____

____

____

____

____

____

Spørgeskema <u>for den nærmeste pårørende</u>

Navn: ____ **Alder:** ________________

Køn: ☐ Mand **Dit forhold til patienten** (fx barn, hustru): ____________________

☐ Kvinde

---

1. Hvor tilfreds er du med den **<u>information</u>** som du fik **<u>før indlæggelsen</u>**?

☐	☐	☐	☐	☐
Virkelig meget	Meget	Moderat	Lidt	Slet ikke

2. Hvor tilfreds er du generelt med i**<u>ndlæggelsesforløbet</u>**?

☐	☐	☐	☐	☐
Virkelig meget	Meget	Moderat	Lidt	Slet ikke

3. I hvor høj grad føler du, at du havde **<u>mulighed for at besøge patienten</u>**?

☐	☐	☐	☐	☐
I meget høj grad	I høj grad	Moderat	I mindre grad	Slet ikke

4. Hvordan oplever du at **<u>indlæggelseslængden</u>** var?

☐	☐	☐	☐	☐
For lang	Lang	Tilpas	Kort	For kort

5. Hvor tilfreds er du med den **<u>information</u>** som du fik i forbindelse **<u>med udskrivelsen</u>**?

☐	☐	☐	☐	☐
Virkelig meget	Meget	Moderat	Lidt	Slet ikke

6. Hvor **fysisk** anstrengende vurderer du at forløbet var **for patienten**?

☐	☐	☐	☐	☐
Virkelig meget	Meget	Moderat	Lidt	Slet ikke

7. Hvor **psykisk** anstrengende vurderer du at forløbet var **for patienten**?

☐	☐	☐	☐	☐
Virkelig meget	Meget	Moderat	Lidt	Slet ikke

8. **Hvor lang tid** vurderer du, at det tog **for patienten** at være tilbage til optimal tilstand?

☐	☐	☐	☐	☐
< 1 måned	1-3 måneder	3-6 måneder	> 6 måneder	Er ikke kommet sig endnu

---

9. Hvor **fysisk** anstrengende vurderer du at forløbet var **for dig, som pårørende**?

☐	☐	☐	☐	☐
Virkelig meget	Meget	Moderat	Lidt	Slet ikke

10. Hvor **psykisk** anstrengende vurderer du at forløbet var **for dig, som pårørende**?

☐	☐	☐	☐	☐
Virkelig meget	Meget	Moderat	Lidt	Slet ikke

11. **Hvor lang tid** vurderer du at det tog **for dig** at være tilbage til optimal tilstand?

☐	☐	☐	☐	☐
< 1 måned	1-3 måneder	3-6 måneder	> 6 måneder	Jeg er ikke kommet mig endnu

12. Hvad kunne vi have gjort bedre for dig i forløbet? ______________________________

____

____

____

Questionnaire for the patient

Name: ____ **Age:** __________________

Gender: ☐ Male ☐ Female

Social status: ☐ Lives alone ☐ Living together/married ☐ Other: ___________________________

Generel health

13. Please indicate on the scale below how good or bad you assess your health **before you had your new heart valve**. The best health status you can imagine is 100 and the worst health status you can imagine is 0. Please mark with a cross (X) on the scale.

0 10 20 30 40 50 60 70 80 90 100

Worst health — Best health

14. Please indicate on the scale below how good or bad you assess your health **today – after you had your new heart valve**. The best health status you can imagine is 100 and the worst health status you can imagine is 0. Please mark with a cross (X) on the scale.

0 10 20 30 40 50 60 70 80 90 100

Worst health — Best health

Symptoms

15. How do you assess your symptoms **before you had your new heart valve**?

- ☐ I had no limitation of physical activity.
- ☐ I experienced dyspnea and/or fatigue by doing moderately to hard physical activity e.g. walking the stairs to more than the second floor.
- ☐ I experienced dyspnea and/or fatigue by doing ordinary physical activity e.g. walking the stairs to less than the second floor or getting dressed.
- ☐ I experienced dyspnea and/or fatigue in rest.

16. How do you assess your symptoms **today – after you had your new heart valve**?

- ☐ I have no limitation of physical activity.
- ☐ I experience dyspnea and/or fatigue by doing moderately to hard physical activity e.g. walking the stairs to more than the second floor.
- ☐ I experience dyspnea and/or fatigue by doing ordinary physical activity e.g. walking the stairs to less than the second floor or getting dressed.
- ☐ I experience dyspnea and/or fatigue when resting.

Physical health

17. How physically stressful do you assess **the procedure** was for you?

☐ Severe ☐ A lot ☐ Moderate ☐ Mild ☐ Not at all

18. How physically stressful do you assess the **period following the procedure** was for you?

☐ Severe ☐ A lot ☐ Moderate ☐ Mild ☐ Not at all

19. **How long time** did it take you to recover physically from the whole process?

☐ < 1 month ☐ 1-3 months ☐ 3-6 months ☐ > 6 months ☐ I didn't recover physically yet

Mental health

20. How mentally stressful do you assess **the procedure** was for you?

☐ Severe ☐ A lot ☐ Moderate ☐ Mild ☐ Not at all

21. How mentally stressful do you assess the **period following the procedure** was for you?

☐ Severe ☐ A lot ☐ Moderate ☐ Mild ☐ Not at all

22. **How long time** did it take you to recover mentally from the whole process?

☐ < 1 month ☐ 1-3 months ☐ 3-6 months ☐ > 6 months ☐ I didn't recover mentally yet

23. If you had to go through the same procedure **again**, how would you feel about it?

☐ Very good ☐ Good ☐ Neutral ☐ Bad ☐ I wouldn't do it again

24. Other comments:

____

____

____

____

____

____

____

____

____

____

____

____

____

____

____

Questionnaire for the nearest informal caregiver

Name: ____ **Age:** __________________

Gender: ☐ Male ☐ Female **Your relation to the patient** (e.g. child, wife)**:** _____________________

Your assessment of hospitalization and information

13. How satisfied are you with the **information** you received **before admission**?

☐ Very satisfied ☐ Satisfied ☐ Moderately ☐ Unsatisfied ☐ Very unsatisfied

14. How satisfied are you in general with the **hospitalization?**

☐ Very satisfied ☐ Satisfied ☐ Moderately ☐ Unsatisfied ☐ Very unsatisfied

15. To what extent do you feel you had the **opportunity to visit the patient?**

☐ Very great extent ☐ Great extent ☐ Moderately ☐ To a lesser extent ☐ Not at all

16. How do you experience the **length of hospitalization?**

☐ Too long ☐ Long ☐ Good ☐ Short ☐ Too short

17. How satisfied are you with the **information** you received at **discharge?**

☐ Very satisfied ☐ Satisfied ☐ Moderately ☐ Unsatisfied ☐ Very unsatisfied

Your assessment of the patient

18. How **<u>physically</u>** stressfull do you assess the whole process was **<u>for the patient</u>**?

☐ Severe ☐ A lot ☐ Moderate ☐ Mild ☐ Not at all

19. How **<u>mentally</u>** stressfull do you assess the whole process was **<u>for the patient</u>?**

☐ Severe ☐ A lot ☐ Moderate ☐ Mild ☐ Not at all

20. **<u>How long time</u>** do you assess it took **<u>for the patient</u>** to be back in optimal condition?

☐ < 1 month ☐ 1-3 months ☐ 3-6 months ☐ > 6 months ☐ Did not recover yet

Your assessment of yourself as a relative

21. How **<u>physically</u>** stressfull do you assess the whole process was **<u>for you, as a relative</u>?**

☐ Severe ☐ A lot ☐ Moderate ☐ Mild ☐ Not at all

22. How **<u>mentally</u>** stressfull do you assess the whole process was **<u>for you, as a relative</u>?**

☐ Severe ☐ A lot ☐ Moderate ☐ Mild ☐ Not at all

23. **<u>How long time</u>** do you assess it took **<u>for you</u>** to be back in optimal condition?

☐ < 1 month ☐ 1-3 months ☐ 3-6 months ☐ > 6 months ☐ I didn´t recover yet

24. What could we have done better for you in the whole process?________________________

____

____

____
