# Supplementary figures and images for: Patients and informal caregivers' experience of surgical and transcatheter aortic valve replacement: Real‐world data contributing to establish value‐based medicine in Denmark
Source: Clin Cardiol. 2019 Mar 14;42(4):444–51. doi: 10.1002/clc.23166 (PMC6712343; doi:10.1002/clc.23166)

Suppl. Figure 1

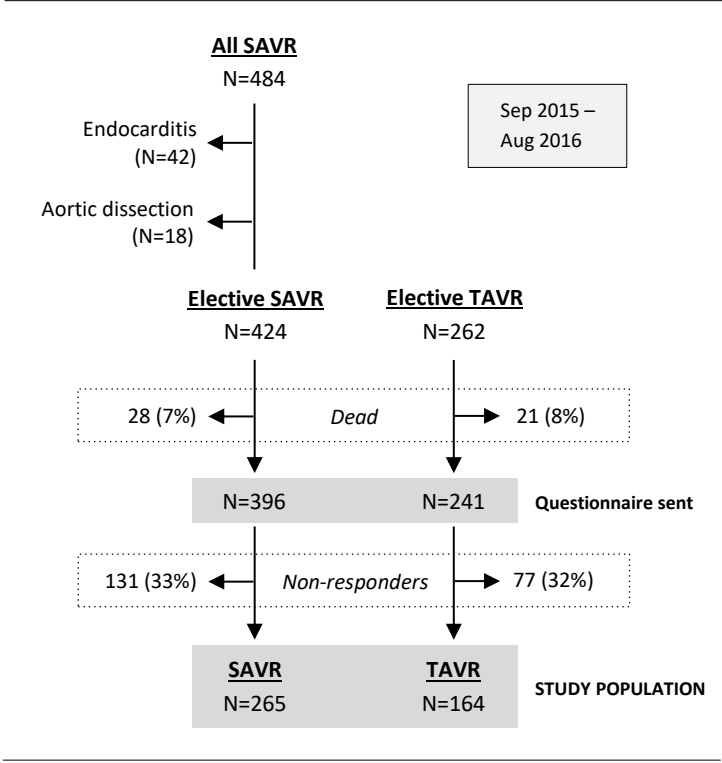

Supplement: Supplementary file 2 — Figure S1. Study design. [file CLC-42-444-s002.pdf]
