# Supplementary material for: Patients and informal caregivers' experience of surgical and transcatheter aortic valve replacement: Real‐world data contributing to establish value‐based medicine in Denmark
Source: Clin Cardiol. 2019 Mar 14;42(4):444–51. doi: 10.1002/clc.23166 (PMC6712343; doi:10.1002/clc.23166)
Supplement: Supplementary file 3 — Figure S2 A and B, Physical and mental stress reported by SAVR patients without concomitant surgical intervention (N = 195). Bar charts indicating the number of patients that felt the procedure and the recovery period physically and mentally stressful to a certain degree (very little, little, moderate, much, very much). In addition, patients indicated how long time it took to recover physically and mentally from the procedure. SAVR, surgical aortic valve replacement. C, Health‐related quality of life (HR‐QoL) in SAVR patients without concomitant cardiac surgery (N = 195). Schematic figure showing the reported HR‐QoL by SAVR patients without concomitant cardiac surgery, before and after intervention. The green‐red bar on the right indicates the number of patients that reported an improved, unchanged or worse HR‐QoL after intervention. SAVR, surgical aortic valve replacement. D and E, Health‐related quality of life (HR‐QoL) in intermediate risk SAVR and TAVR patients (N = 208). Schematic figures showing the reported HR‐QoL by intermediate risk (STS surgical risk score 3‐8%) SAVR and TAVR patients, before and after intervention. The green‐red bar on the right indicates the number of patients that reported an improved, unchanged or worse HR‐QoL after intervention. SAVR, surgical aortic valve replacement; TAVR, transcatheter aortic valve replacement. [file CLC-42-444-s003.pdf]

Suppl. Figure 2

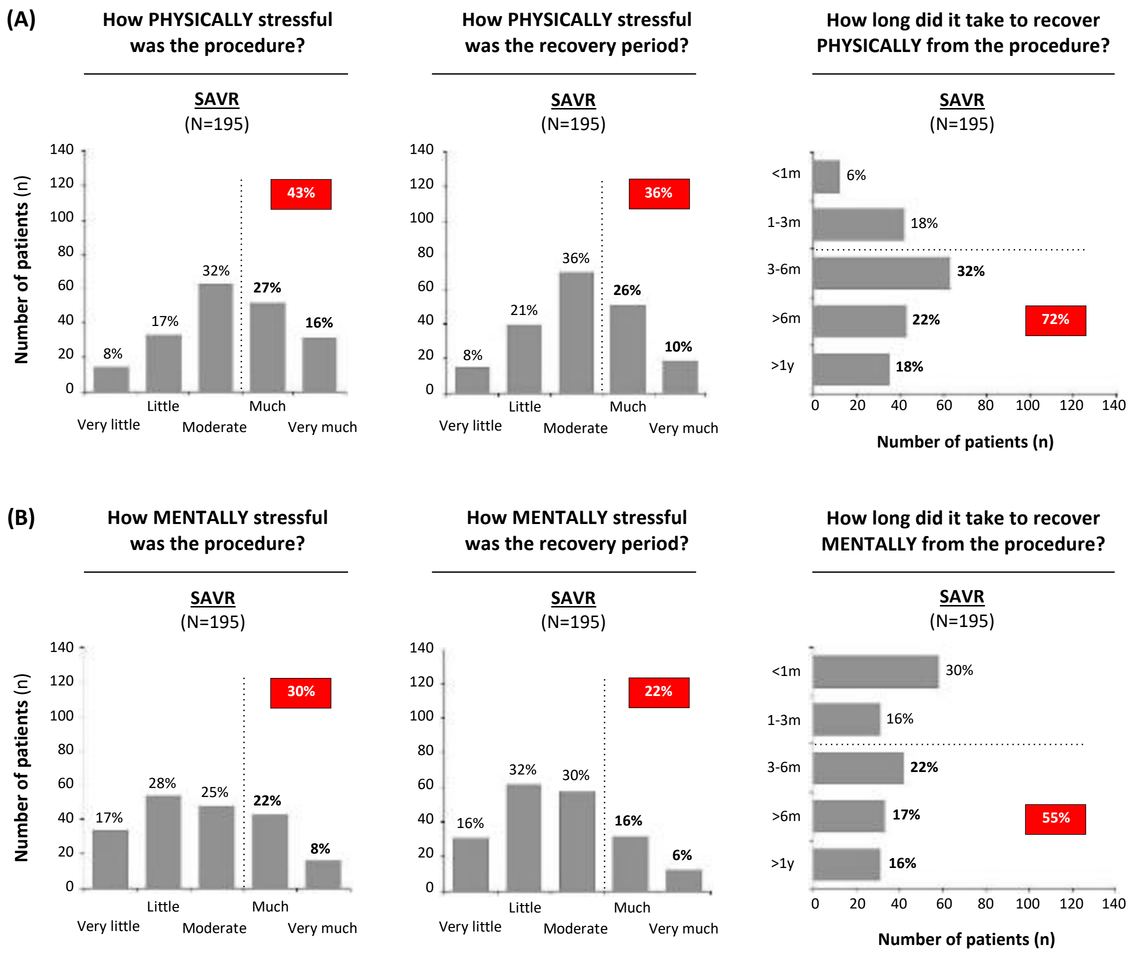

Suppl. Figure 2A-2B. Physical and mental stress reported by SAVR patients without concomitant surgical intervention (N=195). Bar charts indicating the number of patients that felt the procedure and the recovery period physically and mentally stressful to a certain degree (very little, little, moderate, much, very much). In addition, patients indicated how long time it took to recover physically and mentally from the procedure. SAVR, surgical aortic valve replacement.

(C) SAVR without concomitant cardiac surgery (N=195)

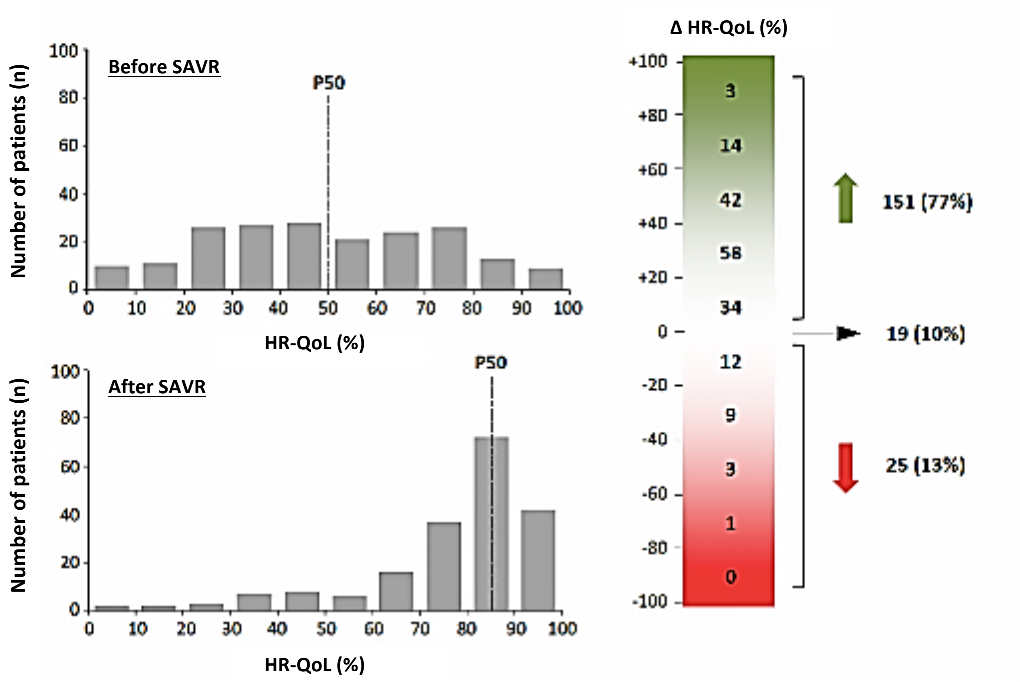

Suppl. Figure 2C. Health-related quality of life (HR-QoL) in SAVR patients without concomitant cardiac surgery (N=195). Schematic figure showing the reported HR-QoL by SAVR patients without concomitant cardiac surgery, before and after intervention. The green-red bar on the right indicates the number of patients that reported an improved, unchanged or worse HR-QoL after intervention. SAVR, surgical aortic valve replacement.

(D) Intermediate risk SAVR patients (N=106)

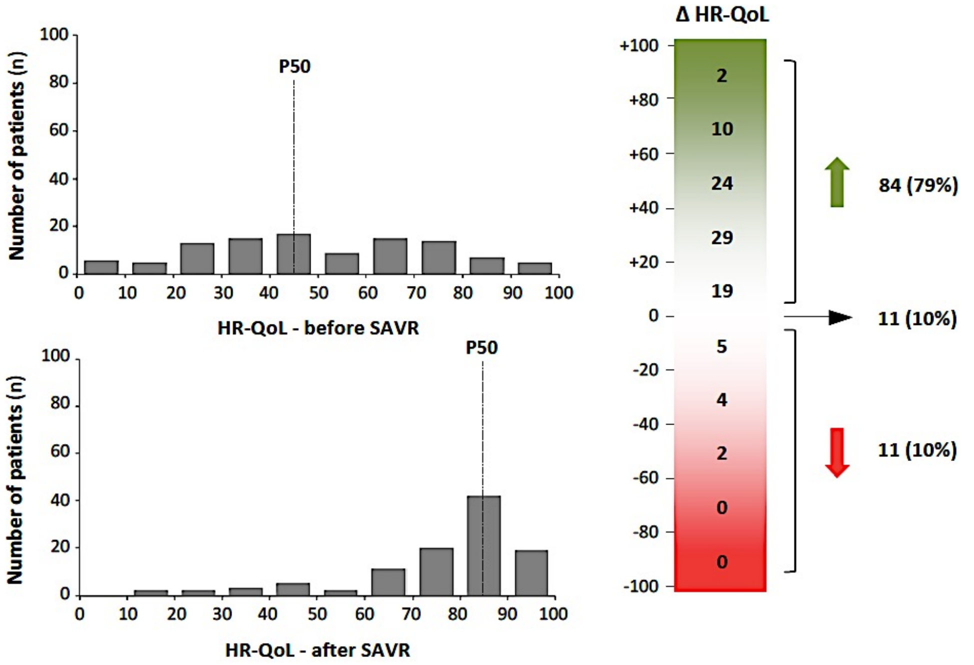

(E) Intermediate risk TAVR patients (N=102)

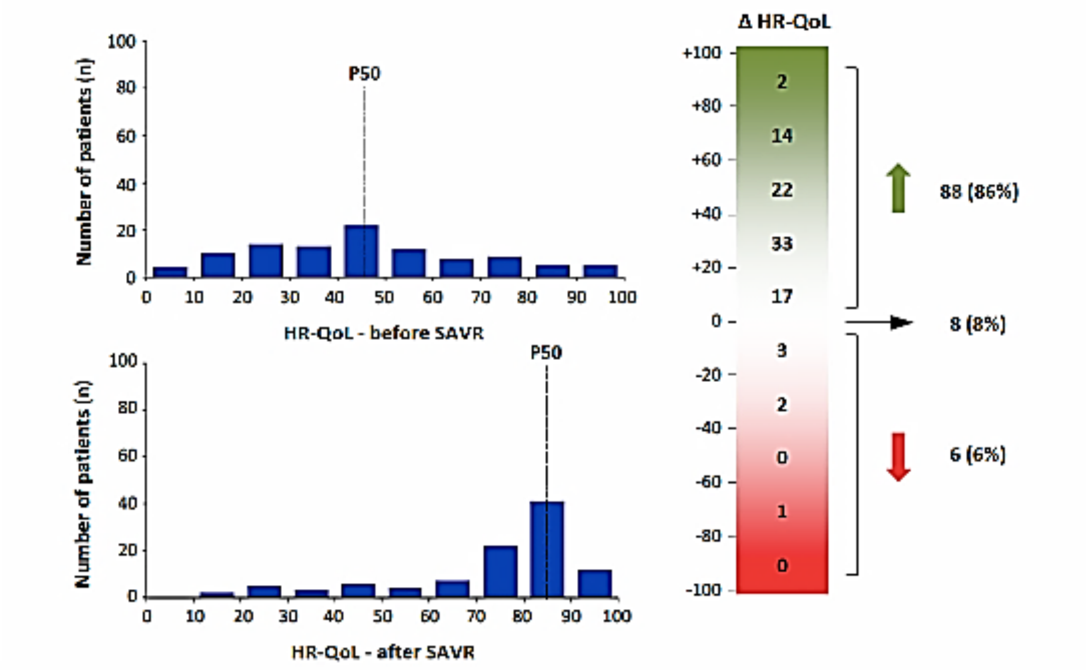

Suppl. Figure 2D-2E. Health-related quality of life (HR-QoL) in intermediate risk SAVR and TAVR patients (N=208). Schematic figures showing the reported HR-QoL by intermediate risk (STS surgical risk score 3-8%) SAVR and TAVR patients, before and after intervention. The green-red bar on the right indicates the number of patients that reported an improved, unchanged or worse HR-QoL after intervention. SAVR, surgical aortic valve replacement; TAVR, transcatheter aortic valve replacement.
