# Supplementary material for: Patients and informal caregivers' experience of surgical and transcatheter aortic valve replacement: Real‐world data contributing to establish value‐based medicine in Denmark
Source: Clin Cardiol. 2019 Mar 14;42(4):444–51. doi: 10.1002/clc.23166 (PMC6712343; doi:10.1002/clc.23166)
Supplement: Supplementary file 4 — Figure S3. NYHA functional classification. Schematic figure showing the reported NYHA functional classification by all SAVR and TAVR patients before and after intervention. The green‐red bar on the right indicates the number of patients that reported an improved, unchanged or worse NYHA classification after intervention, respectively. NYHA, New York Heart Association; SAVR, surgical aortic valve replacement; TAVR, transcatheter aortic valve replacement. [file CLC-42-444-s004.pdf]

Suppl. Figure 3

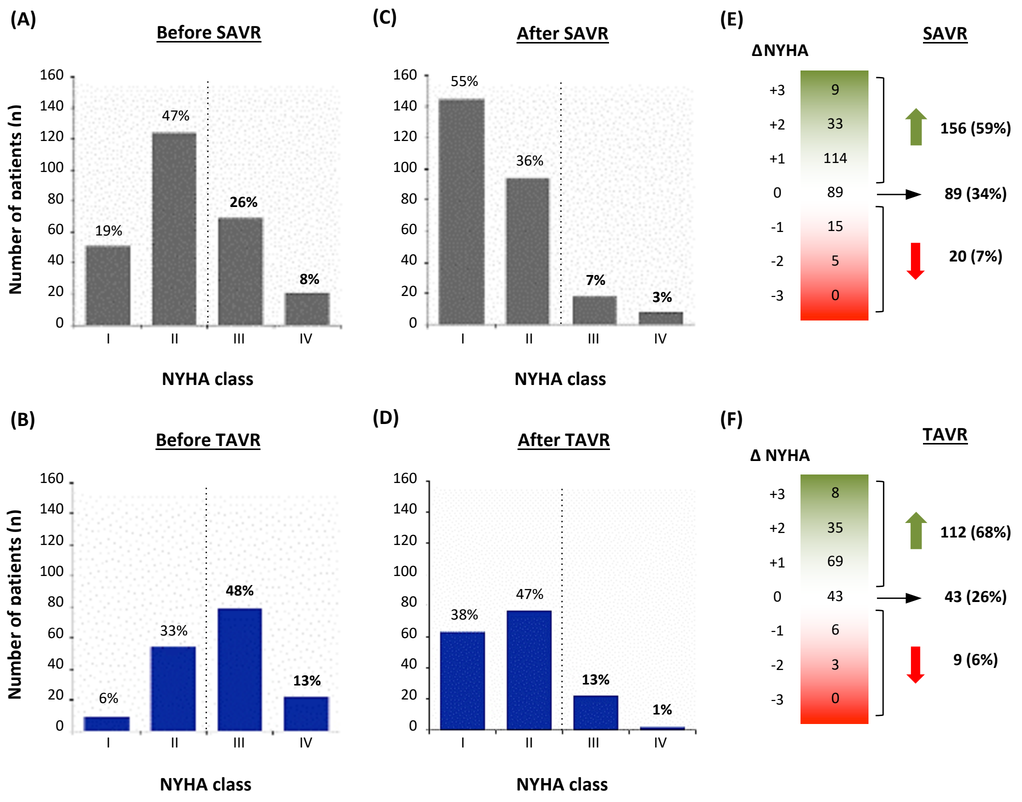

Suppl. Figure 3. NYHA functional classification. Schematic figure showing the reported NYHA functional classification by all SAVR and TAVR patients before and after intervention. The green-red bar on the right indicates the number of patients that reported an improved, unchanged or worse NYHA classification after intervention, respectively. NYHA, New York Heart Association; SAVR, surgical aortic valve replacement; TAVR, transcatheter aortic valve replacement.
