# Supplementary material for: Patients and informal caregivers' experience of surgical and transcatheter aortic valve replacement: Real‐world data contributing to establish value‐based medicine in Denmark
Source: Clin Cardiol. 2019 Mar 14;42(4):444–51. doi: 10.1002/clc.23166 (PMC6712343; doi:10.1002/clc.23166)
Supplement: Supplementary file 5 — Table S1. Baseline characteristics [file CLC-42-444-s005.pdf]

Suppl. Table 1

Suppl. Table 1 Baseline characteristics

	SAVR (N = 265)	TAVR (N=164)	P-Value
**Patient characteristics**			
Age, years	70 ± 10	80 ± 8	< 0.001
Male	193 (73%)	87 (53%)	< 0.001
Social status			
Married / living together	188 (71%)	88 (54%)	< 0.001
Living alone	74 (28%)	69 (42%)	0.004
Other	3 (1%)	7 (4%)	0.078
Arterial hypertension	183 (69%)	118 (72%)	0.597
Diabetes mellitus	42 (16%)	34 (21%)	0.247
Previous myocardial infarction	27 (10%)	13 (8%)	0.540
Previous PCI	16 (6%)	34 (21%)	< 0.001
Previous CABG	3 (1%)	25 (15%)	< 0.001
Peripheral vascular disease	21 (8%)	20 (12%)	0.196
Atrial fibrillation, history	55 (21%)	57 (35%)	< 0.001
Cerebrovascular accident, history	24 (9%)	18 (11%)	0.629
Chronic renal failure (GFR < 60 mL/min)	40 (15%)	46 (28%)	0.02
Chronic lung disease	37 (14%)	21 (13%)	0.845
STS score	3.1 ± 2.9	3.8 ± 2.4	< 0.001
**Echocardiographic assessment**			
LVEF, %	50 ± 12	48 ± 13	0.905
Mean aortic valve gradient, mmHg	43 ± 12	44 ± 14	0.897
Aortic valve area, cm^2	0.7 ± 0.2	0.7 ± 0.2	0.942
Mitral regurgitation ≥ moderate	15 (9%)	13 (8%)	0.470
**Procedural characteristics**			
General anaesthesia	265 (100%)	10 (6%)	< 0.001
Transfemoral approach	-	156 (95%)	-
Aortic valve replacement			
Biological surgical prosthesis	225 (85%)	-	-
Mechanical surgical prosthesis	40 (15%)	-	-
Self-expanding THV	-	100 (61%)	-
Balloon-expandable THV	-	26 (16%)	-
Mechanically-expandable THV	-	38 (23%)	-
Concomitant procedure(s)			
CABG / PCI	79 (30%)	29 (18%)	0.007
Aortic root replacement	54 (20%)	-	-
Other valve	21 (8%)	-	-
**Hospitalization length**			
Intensive care unit, days	2.4 ± 8.1	0.1 ± 0.3	< 0.001
Total length, days	11.2 ± 9.2	3.6 ± 2.4	< 0.001

CABG, coronary artery bypass graft; GFR, glomerular filtration rate; LVEF, left ventricular ejection fraction; PCI, percutaneous coronary intervention; SAVR, surgical aortic valve replacement; STS, Society of Thoracic Surgeons; TAVR, transcatheter aortic valve replacement; THV, transcatheter heart valve.
