# Supplementary material for: Patients and informal caregivers' experience of surgical and transcatheter aortic valve replacement: Real‐world data contributing to establish value‐based medicine in Denmark
Source: Clin Cardiol. 2019 Mar 14;42(4):444–51. doi: 10.1002/clc.23166 (PMC6712343; doi:10.1002/clc.23166)
Supplement: Supplementary file 6 — Table S2. Patients that did not report an improvement in HR‐QoL following intervention [file CLC-42-444-s006.pdf]

Suppl. Table 2 Patients that did not report an improvement in HR-QoL following intervention

	SAVR (N=63)	TAVR (N=27)
Higher degree of dyspnea than before intervention +/- LVEF reduction	8	4
Still unhappy about suboptimal logistics (e.g. hospitalized in 4 different hospitals, too short/long time between diagnosis and intervention, bad communication, etc.)	6	3
Stroke with neurological impairment	3	2
Invalidating episodes of atrial fibrillation	3	-
Mental changes after surgery	3	-
More fatigue than before surgery	2	2
Permanent renal impairment after surgery	2	-
Complaints about mechanical heart valve that can be heard	2	-
Cold/tingling fingers	2	1
Mentally not possible to recover from this major surgery	2	-
Recurrent re-hospitalizations after surgery	2	2
Complaints about need for anticoagulation after surgery	2	-
Dizziness	2	3
Multiple TIA's after surgery	1	-
Diffuse muscle pains after surgery	1	-
Headache in the evenings before falling asleep	1	-
Recurrent infection problems after surgery	1	-
Sternum-infection	1	-
Diaphragmatic paralysis	1	-
Sleeping disorder since surgery	1	-
Recurrent anemia with transfusion need since surgery	1	-
Claudicatio symptoms since intervention	-	1
Much longer time to recover – still not recovered	-	1
Unknown	16	8

HR-QoL, health-related quality of life; LVEF, left ventricular ejection fraction; SAVR, surgical aortic valve replacement; TAVR, transcatheter aortic valve replacement; TIA, transient ischemic attack.
